# DUAL FUNCTIONALITY IN LATER LIFE

**DOI:** 10.1093/geroni/igac059.1248

**Published:** 2022-12-20

**Authors:** Kenneth Ferraro

**Affiliations:** Purdue University, West Lafayette, Indiana, United States

## Abstract

Longevity and quality of life are core interests in gerontology, but debate has ensued as scholars have sought to integrate the two. I propose the concept of dual functionality to examine how humans reach advanced ages while maintaining both physical and cognitive function. Using a large national sample, my colleagues and I operationalize dual functionality and identify life course factors that predict it. Analyses of 33,310 respondents 50 years or older from the Health and Retirement Study show an estimated median age of 74 for loss of dual functionality. Lifetime stress exposure leads to earlier loss of dual functionality, even after adjustment for socioeconomic status and lifestyle factors. Estimates of dual-function life expectancy, moreover, reveal greater racial-ethnic disparities than those for life expectancy per se. Dual functionality may be useful for assessing the quality of longevity across societies and social categories and for identifying exceptional longevity.

